# Operational Domains Governing Melt Flow Index Variability in Industrial Polypropylene Production

**DOI:** 10.3390/polym18131670

**Published:** 2026-07-06

**Authors:** Joaquín Hernández-Fernández, Juan López-Martínez

**Affiliations:** 1 Chemistry Program, Department of Natural and Exact Sciences, University of Cartagena, San Pablo Campus, Cartagena de Indias 130015, Colombia; 2 Department of Natural and Exact Science, Universidad de la Costa, Barranquilla 080002, Colombia; 3 Institute of Materials Technology (ITM), Universitat Politecnica de Valencia (UPV), Plaza Ferrandiz and Carbonell S/N, 03801 Alcoy, Spain

**Keywords:** polypropylene, melt flow index, gas-phase polymerization, multivariate analysis, principal component analysis, reactor fouling, process–quality relationships, Ziegler–Natta catalysts

## Abstract

Maintaining a stable melt flow index (MFI) is a critical objective in industrial polypropylene production because MFI directly reflects polymer molecular weight and strongly influences downstream processing performance. Although the effects of catalyst formulation and hydrogen concentration on polypropylene properties are well established, the operational origins of residual fluctuations in MFI under highly stable industrial conditions remain poorly understood. In this work, the relationships between feedstock quality, process operation, and residual MFI variability were investigated during the production of a commercial polypropylene grade in an industrial gas-phase reactor. A dataset comprising 61 industrial observations was assembled by integrating laboratory quality measurements with operational variables related to hydrogen concentration, catalyst management, reactor hydrodynamics, thermal behavior, productivity, and fouling. In parallel, the concentrations of key catalyst inhibitors, including carbon oxides, sulfur compounds, water, oxygen, acetylene, methylacetylene, propadiene, arsine, and phosphine, were quantified before and after the use of a modified zeolite-based purification system. The purification process reduced catalyst poisons to ppb levels, producing polymer-grade propylene with monomer purity exceeding 99.95 wt.%. Under these highly controlled conditions, the production campaign exhibited remarkable quality stability, with an average MFI of 3.03 g/10 min and a coefficient of variation of only 6.63%. Principal component analysis revealed that two dominant operational domains could describe 86.49% of the total process variability. The first domain was associated with reactor hydrodynamics, fouling behavior, and thermal conditions, whereas the second domain was governed by catalyst-system variables and hydrogen-mediated chain-transfer mechanisms. Variable importance in projection analysis identified Plate Fouling Factor (VIP = 2.17), Production Rate (VIP = 1.33), and H_2_/C_3_ Ratio (VIP = 1.17) as the variables most strongly associated with residual MFI fluctuations. The results demonstrate that once feedstock-related disturbances are effectively minimized, residual MFI variability arises from interactions among the hydrodynamic, thermal, and catalytic operational domains rather than from a single controlling parameter. These findings provide new insights into process–quality relationships in industrial polypropylene manufacturing and establish a practical framework for identifying the operational origins of subtle fluctuations in polymer quality in highly stabilized production systems.

## 1. Introduction

The melt flow index is the most critical quality parameter in the global polypropylene industry, serving as the primary proxy for the polymer’s average molecular weight and rheological behavior. In downstream processing, the MFI dictates the success of high-speed manufacturing techniques such as injection molding, thin-wall extrusion, and fiber spinning [1] (see Table 1). Precise control over the MFI is essential because even minor fluctuations can significantly alter the “die swell” and the mechanical integrity of the final product, leading to processing inconsistencies for the end user. Consequently, maintaining a stable MFI within narrow specifications is the primary objective of industrial polymerization control [2]. In modern industrial gas-phase reactors, the MFI is primarily governed by a complex “chemical recipe” in which hydrogen (H_2_) serves as the dominant chain-transfer agent, terminating polymer growth [3,4]. The catalyst system further refines this mechanism, specifically the ratio of triethylaluminum to external silane donors, which modifies the sensitivity of the active titanium sites to hydrogen [5]. Classical kinetic models established by researchers such as Ray and Kissin emphasize that while reactor temperature and monomer concentration set the baseline for propagation, the H_2_/C_3_ molar ratio ultimately defines the polymer’s fluidity [6]. Despite these established control mechanisms, industrial production is frequently destabilized by trace-level impurities known as catalyst inhibitors, or “poisons.” Components such as carbon monoxide (CO), carbon dioxide (CO_2_), and oxygen (O_2_) are aggressive deactivators that compete with the propylene monomer for active coordination sites. Similarly, pnictogens like arsine and sulfur species such as hydrogen sulfide (H_2_S) and carbonyl sulfide (COS) can severely reduce catalytic productivity and shift the molecular weight distribution, even at parts-per-billion (ppb) concentrations [7]. These inhibitors, often summarized in catalyst poison tables, represent the primary source of “kinetic noise” that prevents the attainment of a perfectly steady state. To mitigate these disturbances, industrial plants employ advanced purification stages, including adsorption beds and modified zeolite catalytic systems, to reach “polymer grade” propylene specifications (>99.95 wt.%). Once these major chemical perturbations are eliminated, the polymerization process enters a regime of high stability. However, even under these refined conditions, a “residual variability” in the MFI often persists, appearing as a multivariate phenomenon rather than a simple univariate drift.

Unlike previous studies that primarily focused on manipulating hydrogen concentration, catalyst formulation, reactor temperature, or kinetic modeling to achieve target melt flow index values, the present work investigates the origin of residual MFI variability under normal industrial operating conditions. Using plant-wide operational data from a commercial gas-phase polypropylene reactor, this study identifies the multivariate process domains associated with MFI fluctuations that persist even when the process remains within specification limits. Furthermore, the integration of PCA, correlation analysis, and VIP ranking provides a holistic process-level interpretation that extends beyond conventional single-variable approaches commonly reported in the literature. This research utilizes principal component analysis and variable Importance in Projection scores to investigate these residual fluctuations, providing evidence that stable industrial production is characterized by distinct “operational domains” driven by the subtle interplay between reactor hydrodynamics and catalytic chemistry.

## 2. Materials and Methods

### 2.1. Industrial Setting and Materials

The study was conducted in a commercial gas-phase polypropylene plant (Esenttia S.A., Cartagena, Colombia) utilizing a high-yield MgCl_2_-supported Ziegler–Natta catalyst provided by the industrial partner (Cartagena, Colombia). The production focused on a specific high-performance grade, ESENTTIA 03H83-AV (Esenttia S.A., Cartagena, Colombia), to ensure consistency in the molecular weight distribution and rheological requirements. The primary monomer, propylene, was purified using a customized catalytic system based on modified zeolites provided by the industrial partner (Cartagena, Colombia) to achieve “polymer grade” specifications (>99.95 wt.%).

### 2.2. Quantification of Catalytic Inhibitors

The chemical composition of the propylene stream was characterized before and after the modified zeolite purification system using a combination of chromatographic and spectroscopic techniques routinely employed for polymer-grade propylene certification. Hydrocarbon impurities, including propane, ethane, ethylene, butenes, acetylene, methylacetylene, and propadiene, were quantified using gas chromatography equipped with a flame ionization detector (GC-FID; 7890B GC System, Agilent Technologies, Santa Clara, CA, USA). Compound identification was verified through gas chromatography–mass spectrometry (GC-MS; 7890B GC System coupled to a 5977B GC/MSD, Agilent Technologies, Santa Clara, CA, USA) when required. Permanent gases, including carbon monoxide (CO), carbon dioxide (CO_2_), and oxygen (O_2_), were determined using gas chromatography coupled with pulsed discharge helium ionization detection (GC-PDHID; 7890B GC System, Agilent Technologies, Santa Clara, CA, USA, equipped with a D-3-I-HP pulsed discharge helium ionization detector, VICI Valco Instruments Co. Inc., Houston, TX, USA), allowing quantification at low-ppm and sub-ppm levels. Sulfur-containing compounds, including hydrogen sulfide (H_2_S), carbonyl sulfide (COS), and total sulfur species, were quantified using gas chromatography equipped with sulfur chemiluminescence detection (GC-SCD; 7890B GC System equipped with an 8355 Sulfur Chemiluminescence Detector, Agilent Technologies, Santa Clara, CA, USA). Water concentration was determined by coulometric Karl Fischer titration (899 Coulometer, Metrohm AG, Herisau, Switzerland) in accordance with industrial quality-control procedures for polymer-grade propylene. Arsine (AsH_3_) and phosphine (PH_3_) were quantified using inductively coupled plasma mass spectrometry (ICP-MS; 7900 ICP-MS, Agilent Technologies, Santa Clara, CA, USA) and complementary gas chromatographic methods when required. Chromatographic data acquisition and processing were performed using OpenLab CDS, version 2.7 (Agilent Technologies, Santa Clara, CA, USA), while GC-MS and ICP-MS data were processed using MassHunter Workstation Software, version 10.1 (Agilent Technologies, Santa Clara, CA, USA). Karl Fischer titration data were processed using tiamo, version 3.0 (Metrohm AG, Herisau, Switzerland). The analytical methodology was selected to ensure reliable quantification of catalyst poisons at concentration levels relevant to industrial polypropylene polymerization. Typical analytical detection limits ranged from the low-ppm level for hydrocarbon impurities to the low-ppb level for sulfur compounds, arsine, phosphine, and permanent gases.

### 2.3. Dataset Acquisition and Variables

The dataset was constructed by integrating laboratory quality control results with high-frequency operational data from the plant’s distributed control system (see Table 2).

All variables included in the multivariate analysis are quantitative. Operational variables were obtained directly from the plant Distributed Control System (DCS), whereas the melt flow index (MFI) was determined by routine laboratory quality control testing in accordance with ASTM D1238 [19]. The Plate Fouling Factor and Cooler Fouling Factor are dimensionless operational indicators generated by the plant DCS to monitor the relative fouling condition of the reactor distributor plate and recycle gas cooler, respectively.

#### Synchronization of Laboratory and DCS Data

**Figure 1:**
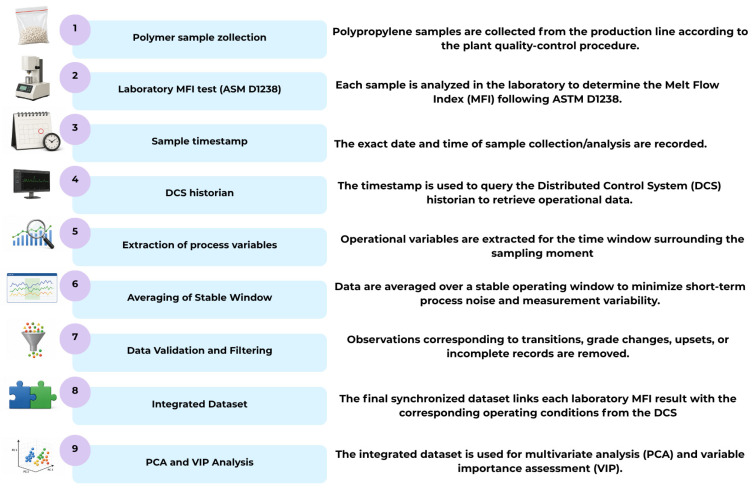
Data synchronization workflow between laboratory MFI measurements and DCS operating data.

Each MFI measurement was associated with its corresponding stable production period. Polymer samples were collected as part of the routine quality-control program and analyzed in accordance with ASTM D1238. For each laboratory result, operational variables were retrieved from the plant distributed control system (DCS) historian and averaged over the representative steady-state production interval associated with the corresponding polymer sample. This approach was adopted to minimize the influence of short-term process fluctuations and measurement noise. Records associated with reactor transitions, operational disturbances, grade changes, start-up or shutdown conditions, and incomplete datasets were excluded from the analysis. After data screening and synchronization, a final dataset comprising 61 matched observations was obtained, with each observation consisting of a laboratory-determined MFI value and the corresponding set of averaged process variables used in the multivariate analysis. The complete synchronization workflow is illustrated in Figure 1, while the detailed synchronization procedure is summarized in Table 3.

### 2.4. Data Processing and Statistical Strategy

The data processing followed a structured workflow to minimize noise and highlight the underlying operational domains (see Table 4).

#### 2.4.1. Data Preprocessing

An initial set of 66 samples was reduced to 61 by removing observations with incomplete time-stamped data or those from reactor transitions. Because the variables have different units (e.g., wt.%, ppm, kg/g), autoscaling was applied. This process involves mean-centering and scaling each variable by its standard deviation, ensuring that each parameter contributes equally to the multivariate model regardless of its magnitude.

#### 2.4.2. Principal Component Analysis

PCA was employed to reduce the dimensionality of the 13 operational variables. By transforming the original variables into orthogonal principal components, we were able to visualize the “operational domains” and identify which clusters of data points corresponded to stable versus variable MFI production [20].

Although the final dataset comprised 61 observations, this sample size was considered suitable for the study’s exploratory multivariate purpose. The analysis included 13 operational variables, giving an observation-to-variable ratio of approximately 4.7. This ratio was considered adequate because PCA was used as an exploratory dimensionality-reduction technique to identify dominant latent operational patterns within a single stabilized industrial production campaign, rather than to develop a universal predictive model. In addition, the first two principal components explained 86.49% of the total variance, indicating that the main multivariate structure of the dataset was effectively captured in a reduced-dimensional space. Therefore, the dataset was sufficient to identify the dominant operational domains within the analyzed campaign. However, the results should be interpreted as campaign-specific and require external validation using additional polypropylene grades, longer monitoring periods, and independent production datasets.

#### 2.4.3. Variable Importance in Projection

To quantify the impact of each variable on the residual MFI variability, a partial least squares framework was used to calculate VIP scores. Variables with a VIP score greater than 1.0 were considered highly influential [21]. This method allowed us to distinguish between the intentional control variables (H_2_/C_3_) and the “uncontrolled” drivers of noise (like the Plate Fouling Factor).

#### 2.4.4. Correlation and Network Analysis

Pearson correlation coefficients were calculated to assess the linear strength of the relationship between individual variables and the MFI. These relationships were then mapped onto a network visualization to illustrate the connectivity between the thermal, chemical, and physical engines of the reactor.

## 3. Results and Discussion

### 3.1. Quantification of Ziegler–Natta Catalyst Inhibitors Before and After Their Adsorption in Modified Zeolite Catalytic Systems

The data presented in Table 5 show the impurity profile of the propylene stream before and after purification using the modified zeolite-based system. All impurity measurements were performed in quintuplicate (*n* = 5) as part of the routine industrial quality-control program. Analytical repeatability was characterized by relative standard deviations (RSDs) below 3% for all monitored species. Values reported using the “<” notation indicate concentrations below the analytical detection limit of the corresponding method. The purification process markedly reduced the concentration of the main impurity classes relevant to Ziegler–Natta catalyst performance. Propane decreased from 1.20 wt.% to 0.03 wt.%, corresponding to 97.5% removal. Although propane is not a catalyst poison, its reduction helps maintain a higher concentration of propylene in the reactor feed. Ethylene decreased by more than 96.0%, which is relevant because residual ethylene may act as an unintended comonomer, thereby modifying the final polymer microstructure. The unsaturated C_3_ impurities propadiene and methylacetylene were reduced by more than 99.3% and 99.1%, respectively, while acetylene decreased by more than 97.5%, reaching a post-purification concentration below 0.05 ppm. These results indicate that the purification stage effectively reduced highly reactive unsaturated impurities that may interfere with catalyst activity and polymerization stability. Oxygenated and permanent-gas impurities were also reduced to trace levels. CO and CO_2_ decreased by more than 95.0%, reaching concentrations below 50 ppb and 100 ppb, respectively. Water decreased by more than 96.6%, reaching a concentration below 100 ppb. These reductions are relevant because CO, CO_2_, O_2_, and H_2_O are commonly associated with active-site inhibition, cocatalyst consumption, and catalyst deactivation in Ziegler–Natta polymerization systems. Therefore, their decrease after purification indicates that a major source of feedstock-related catalyst disturbance was substantially minimized before evaluating residual MFI variability. Sulfur- and pnictogen-containing species showed a similar trend. H_2_S and COS decreased by more than 90.0% and 95.0%, respectively, while total sulfur compounds were reduced to below 50 ppb. Arsine and phosphine were reduced by approximately 90.0%, reaching post-purification concentrations below 1 ppb. These results are relevant because sulfur compounds, arsine, and phosphine are among the most critical trace inhibitors for Ziegler–Natta catalysts, even at ppb-level concentrations. Thus, the observed decrease in these compounds supports the interpretation that the analyzed production campaign was conducted under highly purified feedstock conditions [22].

Overall, the impurity profile after purification indicates that the modified zeolite-based system reduced the main catalyst inhibitors to low-ppm, sub-ppm, or ppb-level concentrations depending on the compound class. This result is important to the study’s objective because it reduces the likelihood that major feedstock-quality excursions dominated residual MFI fluctuations. Instead, the purified-feedstock condition provides an appropriate industrial scenario to evaluate whether the remaining MFI variability was associated with operational variables such as hydrogen response, catalyst productivity, production rate, reactor hydrodynamics, thermal control, and fouling indicators.

Consequently, Table 5 should be interpreted not only as evidence of purification performance, but also as the analytical basis for the subsequent multivariate analysis. By minimizing large impurity-related disturbances, the dataset enabled examination of the residual variability of MFI within a stabilized production window. Under these conditions, PCA and VIP analyses were used to identify the operational domains statistically associated with subtle MFI fluctuations, rather than to attribute the observed variability to a single impurity or isolated process variable.

### 3.2. Production Campaign Stability

The results presented in Table 6 and Table 7 summarize the stability of the selected industrial production campaign and the variability in the main operational variables included in the multivariate analysis. The campaign exhibited an average MFI of 3.03 g/10 min, with a standard deviation of 0.20 g/10 min and a coefficient of variation of 6.63%. This indicates that the production period was sufficiently stable to evaluate residual MFI fluctuations, although the MFI was not completely invariant. Therefore, the dataset represents a stabilized industrial operating window suitable for investigating subtle process–quality relationships.

The operational variables showed different levels of variability during the campaign. The H_2_/C_3_ ratio had a mean of 7.35 and a range of 2.202–12.035, indicating that hydrogen concentration was among the most actively adjusted variables during the production period. This behavior is consistent with hydrogen’s role as the primary chain-transfer agent that regulates polypropylene molecular weight and, consequently, MFI. However, in the present study, the variation in H_2_/C_3_ should be interpreted as part of the operational control strategy rather than as an isolated explanation for MFI variability.

Catalyst-system variables also showed different degrees of dispersion. The TEAL/Ti ratio remained relatively stable, with a standard deviation of 0.252, indicating that it remained within a narrow operating range throughout the campaign. In contrast, the SCA/Ti ratio showed a larger variation, with a standard deviation of 4.19. This indicates that the donor-related catalytic environment varied more substantially than the cocatalyst-to-titanium ratio. Because external donors can affect the hydrogen response of Ziegler–Natta active sites, the observed variability in SCA/Ti may contribute to the multivariate structure captured in the subsequent PCA analysis.

Overall, the descriptive statistics indicate that the campaign exhibited a relatively narrow MFI distribution, with measurable variability in several operational descriptors, particularly the H_2_/C_3_ ratio, SCA/Ti ratio, TEAL/SCA ratio, and catalyst productivity. This contrast is relevant because it suggests that MFI stability was maintained under dynamic operating conditions rather than under completely constant process settings. Accordingly, the dataset provides a suitable basis for applying PCA and VIP analysis to identify which operational domains were statistically associated with residual MFI fluctuations.

**Figure 2:**
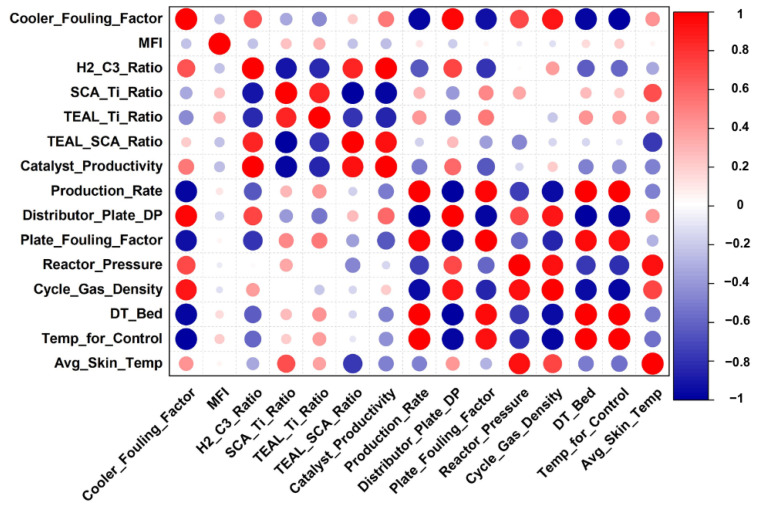
Pearson correlation.

The Pearson correlation heatmap provides an initial view of the relationships between MFI and the operational variables monitored during the production campaign (see Figure 2). The positive association between MFI and the H_2_/C_3_ ratio is consistent with the role of hydrogen as a chain-transfer agent in Ziegler–Natta polypropylene polymerization. However, the magnitude of the individual correlations suggests that MFI variability cannot be explained by hydrogen concentration alone. Other variables, including Temp_for_Control, DT Bed, Avg Skin Temp, Production Rate, and catalyst productivity, also contributed to the operational structure observed during the campaign. The mean catalyst productivity was 39.283 kg/g, with a standard deviation of 17.37 kg/g, indicating that catalyst efficiency varied considerably during the analyzed production period. This variability may reflect the combined influence of catalyst behavior, process adjustments, and reactor operating conditions. Nevertheless, despite dispersion in catalyst productivity and variables such as H_2_/C_3_ and SCA/Ti, the MFI remained within a relatively narrow range, with an average of 3.03 ± 0.20 g/10 min. This contrast suggests that residual MFI variability occurred within a stabilized production window rather than under strongly disturbed operating conditions. The relatively narrow variability of the Cooler Fouling Factor, reported as 0.994 ± 0.028, indicates that this parameter remained stable during the campaign. However, this result should not be interpreted as direct evidence that the purification system prevented fouling. Instead, it suggests that heat-exchanger-related fouling indicators did not exhibit large fluctuations during the period analyzed. Overall, the descriptive statistics and correlation heatmap support the need for a multivariate interpretation, because the residual MFI variability appears to be associated with the combined behavior of chemical, thermal, productivity, and hydrodynamic descriptors rather than with a single operational variable.

### 3.3. Principal Component Analysis

**Figure 3:**
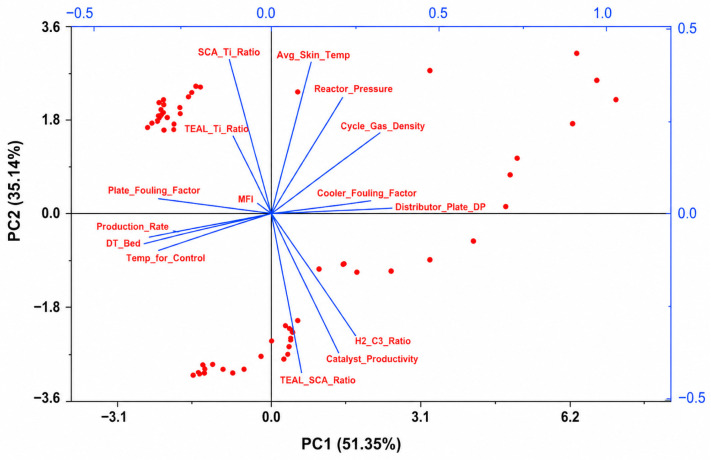
PCA scores plot showing individual process observations as red dots in the PC1–PC2 space. Blue vectors represent variable loadings and indicate the direction of maximum contribution of each process parameter to the principal components.

**Figure 4:**
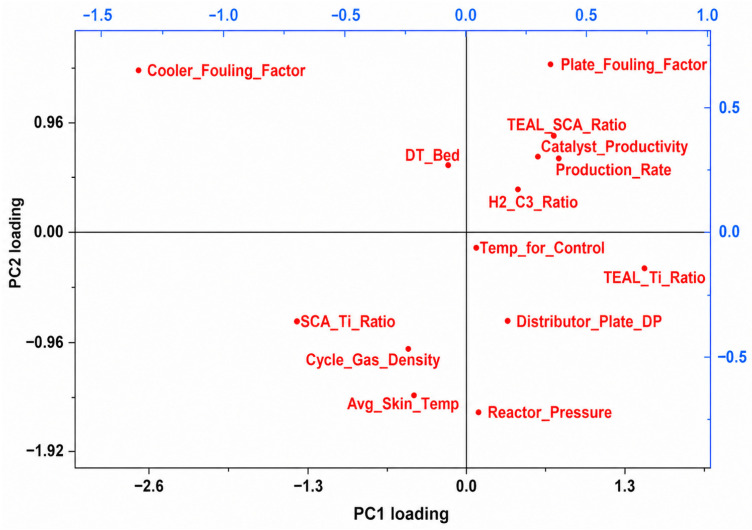
PCA loadings plot.

**Figure 5:**
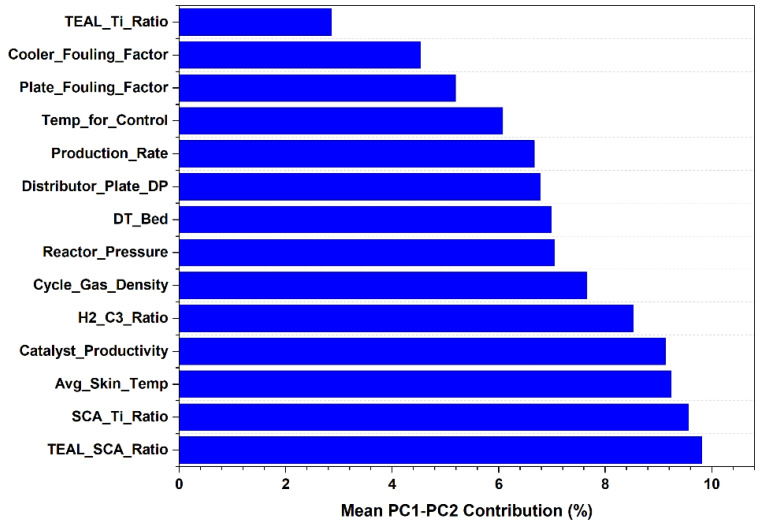
Variable contributions to PCA.

The principal component analysis results presented in Table 8 and Table 9, together with the score and loading plots shown in Figure 3, Figure 4 and Figure 5, summarize the main latent structure of the industrial dataset. The first two principal components explained 86.49% of the total variance, with PC1 accounting for 51.35% and PC2 for 35.14%. This high cumulative explained variance indicates that most of the operational variability observed during the production campaign was concentrated in two principal components, supporting the use of PCA as an exploratory tool for identifying dominant operational domains. The loading structure shown in Table 9 and Figure 4 indicates that PC1 was mainly associated with variables related to reactor operation, hydrodynamics, and thermal management. The strongest contributions to PC1 were observed for Production Rate (−0.360), DT Bed (−0.365), Temp_for_Control (−0.334), Distributor Plate DP (0.368), Cycle Gas Density (0.324), Plate Fouling Factor (−0.319), and Cooler Fouling Factor (0.299). Therefore, PC1 can be interpreted as a physical–thermal operational domain associated with production intensity, gas distribution, heat-removal conditions, and fouling-related indicators. In contrast, PC2 was mainly associated with catalyst-system and hydrogen-response variables. The highest absolute loadings on PC2 corresponded to TEAL/SCA Ratio (0.434), SCA/Ti Ratio (−0.419), Avg Skin Temp (−0.413), Catalyst Productivity (0.379), H_2_/C_3_ Ratio (0.330), and Reactor Pressure (−0.312). This loading pattern suggests that PC2 reflects variations in catalyst chemistry, donor/co-catalyst balance, hydrogen-mediated chain-transfer conditions, and catalyst productivity. Therefore, the PCA results indicate that the industrial process variability was structured around two main domains: a physical–thermal domain, represented by PC1, and a catalyst/hydrogen-response domain, represented by PC2.

The PCA score plot in Figure 3 illustrates the distribution of the 61 observations in this latent space. The dispersion of the samples along PC1 and PC2 suggests that the reactor did not operate at a single fixed point during the campaign, but within a defined multivariate operating region. Samples located in different areas of the score plot were associated with different combinations of hydrodynamic, thermal, and catalyst-system variables. However, because no formal clustering algorithm was applied, these regions should be interpreted as operational tendencies rather than statistically defined clusters. The color gradient associated with MFI in the PCA score plot suggests that residual MFI variability was not randomly distributed across the latent space. Instead, MFI values appeared to vary with the combined position of the samples along PC1 and PC2. This observation supports the interpretation that residual MFI fluctuations were associated with simultaneous changes in physical–thermal and catalyst-related variables, rather than with a single isolated process parameter. Nevertheless, these patterns should be interpreted as statistical associations within the analyzed campaign and not as direct evidence of causality.

Overall, the PCA results provide a compact representation of the multivariate behavior of the production campaign. The separation of operational information into two dominant components supports the central hypothesis of this study: under stabilized feedstock conditions, residual MFI variability is better interpreted as a multivariate process–quality phenomenon involving reactor hydrodynamics, thermal behavior, fouling indicators, catalyst productivity, and hydrogen response.

Catalyst-system variables and hydrogen concentration mainly governed PC2. The strong loadings associated with the SCA/Ti ratio, TEAL/SCA ratio, catalyst productivity, and H_2_/C_3_ ratio indicate that this component reflects variations in catalyst activity and chain-transfer conditions, which are directly related to molecular-weight development and, consequently, to melt-flow index behavior. Residual MFI fluctuations during stable industrial polypropylene production were governed by two interacting operational domains: (i) reactor hydrodynamics and fouling behavior (PC1), and (ii) catalyst-system chemistry and hydrogen-mediated chain transfer (PC2) (see Figure 5).

### 3.4. Validation of the Multivariate Analysis

Prior to interpretation, the robustness of the multivariate analysis was evaluated using explained variance and component retention criteria. The first principal component (PC1) accounted for 51.35% of the total variance, while PC2 explained an additional 35.14%, resulting in a cumulative explained variance of 86.49% (Table S2 and Figure S1). This level of variance capture indicates that the first two principal components adequately represent the multivariate structure of the industrial dataset and provide a robust basis for process interpretation. The scree plot revealed a marked decrease in explained variance after PC2, supporting the retention of two principal components for subsequent analysis. Variable Importance in Projection (VIP) scores were subsequently used as an exploratory variable-ranking tool to identify the operational parameters most strongly associated with MFI variability. The complete VIP ranking is provided in Table S3 and Figure S2.

### 3.5. Operational Factors Associated with Residual MFI Fluctuations

**Figure 6:**
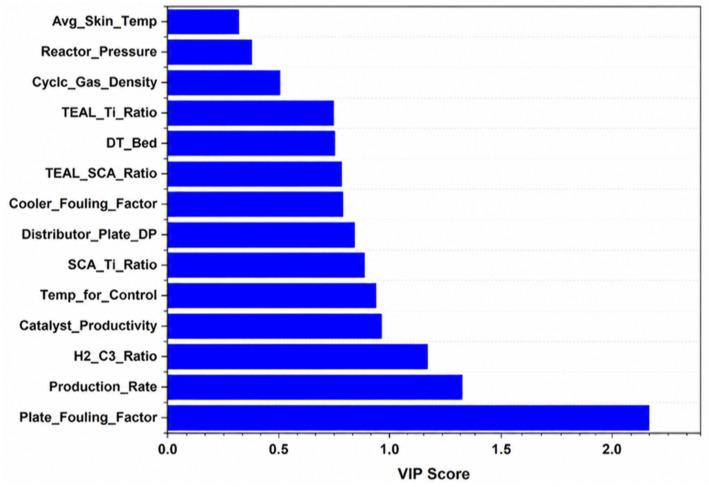
VIP scores for residual MFI fluctuations.

The VIP analysis presented in Table 10 and Figure 6 identified Plate Fouling Factor, Production Rate, and H_2_/C_3_ Ratio as the variables with VIP scores above 1.0, indicating that these variables had the highest relative importance in the PLS model associated with residual MFI variability. Plate Fouling Factor showed the highest VIP score (2.17), followed by Production Rate (1.33) and H_2_/C_3_ Ratio (1.17). These results suggest that, within the analyzed production campaign, residual MFI fluctuations were associated not only with the intentional hydrogen-control variable, but also with physical reactor indicators related to fouling and production intensity.

The high VIP score of Plate Fouling Factor should be interpreted as a statistical association rather than direct evidence of causality. In gas-phase polypropylene reactors, fouling-related indicators may reflect changes in gas distribution, heat-transfer efficiency, local hydrodynamic stability, or solids circulation behavior. Therefore, the ranking of Plate Fouling Factor suggests that this variable contains relevant information about the operating conditions associated with residual MFI variability. However, additional campaigns, independent datasets, or direct hydrodynamic measurements would be required to confirm a mechanistic cause-and-effect relationship. Production Rate was the second most relevant variable according to the VIP ranking. This result suggests that throughput-related changes were associated with residual MFI fluctuations during the analyzed campaign. Variations in production rate may affect residence time distribution, heat removal demand, and reactor thermal balance, all of which are relevant factors in gas-phase polymerization. Nevertheless, this association should not be interpreted as evidence that production rate alone controls MFI. Instead, it indicates that production intensity contributed to the multivariate operational structure captured by the PLS-VIP model. The H_2_/C_3_ ratio also showed a VIP score above 1.0, consistent with its known role as the primary chain-transfer control variable in polypropylene polymerization. However, its VIP score was lower than those of Plate Fouling Factor and Production Rate. This does not reduce the importance of hydrogen in MFI control; rather, it indicates that, under the stabilized conditions of this campaign, residual MFI variability was not explained exclusively by hydrogen dosing. Instead, the results support a multivariate interpretation in which hydrogen response, reactor hydrodynamics, fouling indicators, and production intensity contributed simultaneously to the observed MFI fluctuations. Variables with VIP scores below 1.0, including Catalyst Productivity (0.96), Temp_for_Control (0.94), SCA/Ti Ratio (0.89), Distributor Plate DP (0.84), Cooler Fouling Factor (0.79), TEAL/Ti Ratio (0.71), TEAL/SCA Ratio (0.67), Reactor Pressure (0.54), and Cycle Gas Density (0.48), should not be interpreted as irrelevant to polypropylene polymerization. Rather, their lower VIP values indicate that, within this specific stabilized campaign, they had lower relative importance in explaining residual MFI variability compared with the variables above the VIP threshold.

The VIP results reinforce the interpretation that residual MFI variability under purified-feedstock and stabilized production conditions is a multivariate process–quality phenomenon. The variables most strongly associated with MFI fluctuations were not limited to the chemical recipe; they also included indicators of fouling and production intensity. Therefore, monitoring strategies based only on hydrogen concentration may be insufficient to describe subtle MFI variability under highly stabilized industrial conditions.

### 3.6. Correlation Analysis

**Figure 7:**
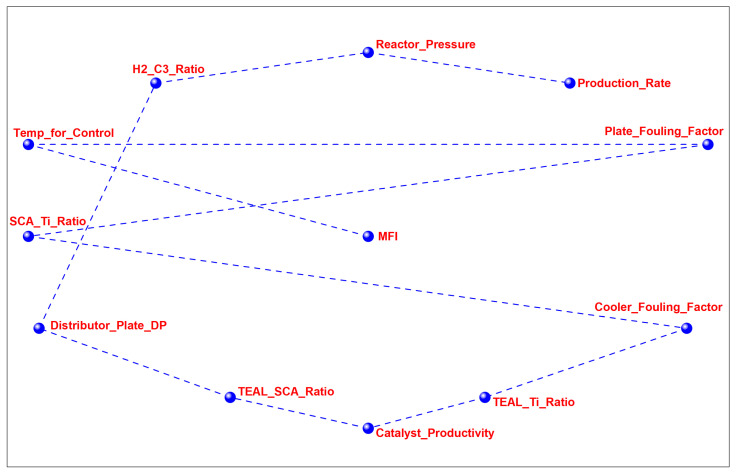
Correlation network of selected operational variables.

The correlation analysis presented in Table 11 and the network visualization shown in Figure 7 provide a complementary univariate perspective on the relationships between MFI and the selected operational variables. In contrast to the PCA and VIP results, which describe multivariate patterns, Pearson correlation coefficients evaluate only linear pairwise relationships. Therefore, this analysis was used to determine whether any individual process variable showed a strong direct linear association with residual MFI variability. The Pearson correlation coefficients between MFI and the operational variables were relatively low. The highest absolute correlations were observed for Temp_for_Control (r = 0.166), Plate Fouling Factor (r = −0.162), SCA/Ti Ratio (r = 0.141), Cooler Fouling Factor (r = −0.140), TEAL/Ti Ratio (r = 0.115), Catalyst Productivity (r = −0.113), and TEAL/SCA Ratio (r = −0.112). These values indicate that no single variable explained residual MFI variability through a strong linear relationship. Therefore, the correlation analysis supports the interpretation that MFI fluctuations during the stabilized campaign were not driven by a single operational parameter. The weak individual correlations do not contradict the PCA and VIP results. Instead, they suggest that residual MFI variability was distributed across several interacting operational descriptors. For example, Plate Fouling Factor showed only a weak negative Pearson correlation with MFI, but it presented the highest VIP score in the PLS-based analysis. This indicates that the variable may provide relevant information in the multivariate model, even if its isolated linear relationship with MFI is limited. Thus, the VIP result should be interpreted as evidence of multivariate relevance, not as proof of a direct linear or causal effect. The network visualization in Figure 7 further illustrates that the operational variables were interconnected within the production campaign. Relationships among Production Rate, DT Bed, H_2_/C_3_ Ratio, Catalyst Productivity, and fouling-related indicators suggest that residual MFI variability occurred within a coupled operational environment involving chemical dosing, catalyst response, thermal behavior, and hydrodynamic indicators. However, these relationships should be interpreted as statistical associations observed during the analyzed campaign rather than as direct mechanistic causality.

These results reinforce the need for a multivariate interpretation of MFI variability under stabilized industrial conditions. The low Pearson coefficients indicate that univariate monitoring alone may be insufficient to describe subtle MFI fluctuations. In contrast, the combined use of PCA, VIP analysis, and correlation mapping provides a more informative framework for identifying operational domains associated with residual variability in process quality.

### 3.7. Comparison with Literature and Industrial Practice

The correlation analysis in Table 12 and the network visualization in Figure 6 provide a complementary perspective for understanding residual melt flow index variability. While the individual Pearson correlation coefficients (r) were relatively low, the multivariate relevance observed in the PCA and VIP analyses suggests that MFI variability in the stabilized industrial process was not associated with a single dominant linear relationship. Instead, the results indicate that residual MFI fluctuations were distributed across several interconnected operational variables. The relatively low Pearson coefficients observed across the operational variables suggest that isolated univariate relationships cannot adequately describe the residual variability in MFI. This finding supports the use of PCA and VIP methodologies to evaluate the combined contribution of multiple operational factors acting simultaneously within the reactor.

The negative correlation between Plate Fouling Factor and MFI should be interpreted as a weak linear association rather than as direct evidence of causality. Its high VIP score (2.17) indicates that this variable contributed relevant information within the multivariate model, even though its isolated Pearson correlation with MFI was low. Therefore, the combined interpretation of Pearson correlation and VIP analysis suggests that fouling-related indicators may contribute to residual MFI variability through multivariate interactions rather than through a simple linear effect. In industrial gas-phase reactors, fouling can be associated with changes in gas distribution, residence-time behavior, and local thermal stability; however, these mechanisms were not directly measured in the present study and should therefore be considered as possible interpretations rather than confirmed causes.

The positive correlation between the SCA/Ti ratio and MFI suggests that the external donor may have contributed to the observed variability, although the magnitude of the correlation was low. External donors are mainly used to control stereospecificity, but they may also influence the hydrogen response of different active-site populations. Similarly, the TEAL/Ti ratio (r = 0.115) showed only a weak positive association with MFI, suggesting that aluminum alkyl levels were not a dominant isolated source of MFI variability within the analyzed operating window. Figure 6 also shows relationships among Production Rate, DT Bed, H_2_/C_3_ Ratio, Catalyst Productivity, and fouling-related indicators, supporting the interpretation that the process operated as an interconnected system rather than as a set of independent variables.

Because the modified zeolite-based purification system reduced most catalyst inhibitors to trace levels, the residual correlations were mainly associated with operational variables rather than with large impurity excursions. However, the low individual r values indicate that no single variable explained the residual 6.63% MFI variability by itself. Therefore, the correlation results should be interpreted together with the PCA and VIP analyses. From an industrial perspective, these findings suggest that multivariate monitoring may provide a more informative description of residual MFI variability than a univariate strategy focused only on hydrogen concentration. In this context, variables such as Plate Fouling Factor, Production Rate, H_2_/C_3_ Ratio, and catalyst productivity may be useful indicators for identifying operating regions associated with subtle MFI fluctuations.

The findings of this study have relevant implications for industrial polypropylene manufacturing. Conventional process optimization strategies often focus primarily on hydrogen concentration because of its direct influence on molecular weight and melt flow index. However, the present results suggest that residual MFI variability under stabilized production conditions cannot be fully described by hydrogen control alone. Once feedstock impurities have been substantially minimized through advanced purification systems, further interpretation of polymer quality consistency requires considering multiple operational domains simultaneously.

The multivariate analysis indicated that reactor hydrodynamics, fouling-related indicators, thermal behavior, catalyst productivity, and hydrogen response were jointly associated with residual MFI fluctuations during the analyzed campaign. In particular, the high VIP score associated with Plate Fouling Factor suggests that physical reactor indicators may contain relevant information about subtle MFI variability when major chemical disturbances have been minimized. From an operational perspective, these results support the inclusion of both chemical and physical process indicators in monitoring strategies for stabilized polypropylene production.

Monitoring the reactor position in the PCA latent space may provide a useful complementary approach for detecting deviations from the typical operating region before large MFI excursions are observed. Nevertheless, this application should be considered exploratory and would require validation with additional grades, longer production periods, and independent industrial datasets. Consequently, the proposed framework should be interpreted as a practical exploratory approach for identifying operational variables associated with product-consistency variations in highly stabilized industrial polypropylene production systems.

## 4. Conclusions

A comprehensive multivariate analysis was performed to investigate the operational variables associated with residual melt flow index (MFI) variability during stable industrial production of polypropylene in a gas-phase reactor. The modified zeolite purification system showed effective removal of catalyst poisons and feedstock contaminants, reducing critical inhibitor concentrations from ppm to ppb levels and producing polymer-grade propylene with a purity greater than 99.95 wt.%. The resulting reduction in feedstock-related disturbances provided a suitable framework for evaluating operational factors associated with fluctuations in residual polymer quality. The analyzed production campaign exhibited a stable quality profile, with an average MFI of 3.03 g/10 min and a coefficient of variation of 6.63%, indicating a controlled but not invariant industrial operation. Under these conditions, principal component analysis indicated that two operational domains accounted for 86.49% of the total process variability. The first domain was mainly associated with reactor hydrodynamics, fouling behavior, and thermal management, whereas the second domain was associated with catalyst-system chemistry, hydrogen response, and catalyst productivity. VIP analysis identified Plate Fouling Factor, Production Rate, and H_2_/C_3_ Ratio as the operational variables with the highest relative importance in relation to residual MFI fluctuations. These results suggest that polymer quality stability is associated not only with catalyst chemistry and hydrogen control, but also with physical reactor indicators related to gas distribution, heat transfer, and fouling development. The weak individual Pearson correlations between process variables and MFI indicate that no single operational parameter showed a strong isolated linear relationship with residual MFI variability. Instead, this variability appeared to be associated with the combined interaction of multiple operational domains acting simultaneously within the reactor. From an industrial perspective, the results suggest that minimizing feedstock impurities is an important first step toward polymer quality stability; however, once catalyst poisons are effectively controlled, further interpretation of MFI consistency should consider reactor hydrodynamics, fouling behavior, thermal stability, and catalyst-system operation. Overall, this study supports the use of multivariate analysis for identifying operational domains associated with subtle process–quality relationships in industrial polypropylene production. Nevertheless, because the dataset corresponds to one polypropylene grade, one reactor, and one stabilized production campaign, the proposed framework should be considered exploratory and requires validation using additional grades, longer monitoring periods, and independent industrial datasets. The dataset corresponds to one commercial polypropylene grade, one industrial gas-phase reactor, and one stabilized production campaign. Therefore, the identified operational domains should be interpreted as grade-specific and plant-specific patterns rather than universal descriptors of polypropylene production.

## Figures and Tables

**Table 1:** Typical Concentrations of Catalyst Poisons in Propylene and Their Reported Impact on Polypropylene Polymerization.

Impurity	Concentration at Which Effects Have Been Reported	Main Effect on Polymerization	Typical Polymer-Grade Specification
H_2_S	0.07–5 ppm	Increase in MFI, decrease in molecular weight, and catalyst productivity [8]	<1 ppm
H_2_S	0.5–1.0 ppm	Significant reduction in molecular weight and increase in flowability [9]	<1 ppm
O_2_	0.5–20.2 ppm	Reduction in catalyst productivity; relatively mild poisoning effect [10]	<5 ppm
CO	0.1–5 ppm	Strong adsorption on active sites; major productivity losses [11]	<1 ppm
CO_2_	0.1–5 ppm	Catalyst inhibition and increased scavenger consumption [12]	<3 ppm
H_2_O	>1–5 ppm	Catalyst deactivation and unstable polymerization [13]	<5 ppm
COS	0.01–1 ppm	Severe catalyst poisoning [14]	<1 ppm
Arsine (AsH_3_)	10–50 ppb	Strong catalyst poisoning and productivity collapse [15]	<20 ppb
Phosphine (PH_3_)	10–50 ppb	Strong catalyst poisoning [16]	<20 ppb
Acetylene	0.15–3.5 ppm	Reduced activity and polymerization disturbances [17]	<5 ppm
Methylacetylene	0.04–0.2 ppm	Catalyst poisoning and selectivity changes [18]	<5 ppm

**Table 2:** Process Variables Included in the Multivariate Analysis.

Category	Variable	Industrial Definition	Type	Source
Gas Composition	H_2_/C_3_ Ratio	Hydrogen-to-propylene molar ratio	Quantitative	DCS
Catalyst System	SCA/Ti Ratio	External donor-to-titanium ratio	Quantitative	DCS
Catalyst System	TEAL/Ti Ratio	Triethylaluminum-to-titanium ratio	Quantitative	DCS
Catalyst System	TEAL/SCA Ratio	Triethylaluminum-to-external donor ratio	Quantitative	DCS
Reactor Performance	Production Rate	Polypropylene production rate	Quantitative	DCS
Reactor Performance	Catalyst Productivity	Catalyst productivity	Quantitative	DCS
Reactor Conditions	Reactor Pressure	Average reactor pressure	Quantitative	DCS
Reactor Conditions	Bed Temperature	Average reactor bed temperature	Quantitative	DCS
Fouling Indicators	Plate Fouling Factor	Plate fouling index	Quantitative	DCS
Fouling Indicators	Cooler Fouling Factor	Cooler fouling index	Quantitative	DCS
Hydrodynamics	Distributor Plate DP	Pressure drop across the distributor plate	Quantitative	DCS
Hydrodynamics	Cycle Gas Density	Recycle gas density	Quantitative	DCS
Thermal Profile	Average Skin Temperature	Average reactor skin temperature	Quantitative	DCS
Polymer Quality	Melt Flow Index	Polymer quality response variable	Quantitative	Laboratory

**Table 3:** Procedure used for synchronization of laboratory MFI measurements and DCS operational data.

Step	Description
Polymer sampling	Samples collected during routine quality-control activities
Laboratory analysis	MFI determined according to ASTM D1238
Sample association	Each MFI value is linked to its corresponding stable production period.
DCS extraction	Process variables retrieved from the plant historian database
Data aggregation	Operational variables averaged over the representative steady-state interval.
Data filtration	Reactor transitions, grade changes, disturbances, and incomplete records removed
Final dataset	61 synchronized observations used for multivariate analysis

**Table 4:** Dataset Characteristics and Data Processing Strategy.

Parameter	Value
Polymer Grade	03H83-AV
Polymerization Process	Industrial gas-phase polypropylene polymerization
Initial Laboratory Samples	66
Final Samples Used for Modeling	61
Response Variable	Melt Flow Index
Data Integration Strategy	Laboratory samples matched with operational data
Missing Data Treatment	Removal of incomplete observations
Data Scaling	Autoscaling (mean-centered and variance-scaled)
Statistical Methods	Correlation analysis, PCA, and VIP analysis
Confidentiality Strategy	Variable anonymization and normalization

**Table 5:** Changes in Ziegler–Natta catalyst inhibitor before and after purification using modified zeolite catalytic systems.

Component	Before Purification	After Purification (Polymer Grade)	Typical Analytical Technique	Potential Impact on Ziegler–Natta Catalysts	n	Analytical Repeatability
Propylene (C_3_H_6_)	98.50 wt.%	99.95 wt.%	GC-FID	Main monomer	5	RSD < 3%
Propane (C_3_H_8_)	1.20 wt.%	0.03 wt.%	GC-FID	Inert diluent	5	RSD < 3%
Ethylene	500 ppm	<20 ppm	GC-FID	Comonomer interference	5	RSD < 3%
Ethane	300 ppm	<10 ppm	GC-FID	Inert	5	RSD < 3%
Butenes	120 ppm	<5 ppm	GC-FID	May alter polymer microstructure	5	RSD < 3%
Propadiene (Allene)	15 ppm	<0.1 ppm	GC-MS/GC-FID	Catalyst poisoning	5	RSD < 3%
Methylacetylene (Propyne)	12 ppm	<0.1 ppm	GC-MS/GC-FID	Catalyst poisoning	5	RSD < 3%
Acetylene	2 ppm	<0.05 ppm	GC-MS/GC-FID	Strong catalyst poison	5	RSD < 3%
Carbon Monoxide (CO)	1 ppm	<50 ppb	GC-PDHID	Active-site deactivation	5	RSD < 3%
Carbon Dioxide (CO_2_)	2 ppm	<100 ppb	GC-PDHID	Catalyst inhibition	5	RSD < 3%
Oxygen (O_2_)	1 ppm	<100 ppb	GC-PDHID/O_2_ Analyzer	Active-site poisoning	5	RSD < 3%
Water (H_2_O)	3 ppm	<100 ppb	Karl Fischer	Catalyst deactivation	5	RSD < 3%
Hydrogen sulfide (H_2_S)	0.10 ppm	<10 ppb	GC-SCD	Severe catalyst poison	5	RSD < 3%
Carbonyl Sulfide (COS)	0.20 ppm	<10 ppb	GC-SCD	Severe catalyst poison	5	RSD < 3%
Total Sulfur Compounds	0.50 ppm	<50 ppb	UV Fluorescence/GC-SCD	Active-site poisoning	5	RSD < 3%
Arsine (AsH_3_)	10 ppb	<1 ppb	ICP-MS	Catalyst poison	5	RSD < 3%
Phosphine (PH_3_)	10 ppb	<1 ppb	GC-PFPD	Catalyst poison	5	RSD < 3%

**Table 6:** Descriptive Statistics of the Selected Production Campaign.

Parameter	Mean	SD	CV (%)
MFI (g/10 min)	3.03	0.20	6.63

**Table 7:** Descriptive Statistics of Operational Variables.

Variable	Mean	SD	CV (%)
Catalyst Productivity	39.283	17.374	44.23
H_2_/C_3_ Ratio	7.350	4.142	56.35
SCA/Ti Ratio	10.458	4.191	40.08
TEAL/SCA Ratio	5.082	1.999	39.34
Production Rate	32.450	4.379	13.49
DT Bed	12.589	2.585	20.53
Cycle Gas Density	64.088	2.024	3.16
Avg Skin Temp	64.697	0.667	1.03
Temp_for_Control	69.539	0.866	1.25
Reactor Pressure	31.228	0.353	1.13
Plate Fouling Factor	1.641	0.036	2.19
Cooler Fouling Factor	0.994	0.028	2.82
Distributor Plate DP	0.176	0.011	6.25
TEAL/Ti Ratio	44.878	0.252	0.56

**Table 8:** Explained Variance of Principal Components.

PC	Explained Variance (%)	Cumulative Variance (%)
PC1	51.35	51.35
PC2	35.14	86.49
PC3	6.85	93.34
PC4	3.55	96.89

**Table 9:** PCA Loadings for the First Two Principal Components.

Variable	PC1	PC2
H_2_/C_3_ Ratio	0.248	0.330
SCA/Ti Ratio	−0.126	−0.419
TEAL/Ti Ratio	−0.114	−0.210
TEAL/SCA Ratio	0.088	0.434
Production Rate	−0.360	0.063
Catalyst Productivity	0.197	0.379
Distributor Plate DP	0.368	−0.012
Plate Fouling Factor	−0.319	−0.041
Cooler Fouling Factor	0.299	−0.035
Reactor Pressure	0.208	−0.312
Cycle Gas Density	0.324	−0.220
DT Bed	−0.365	0.079
Temp_for_Control	−0.334	0.099
Avg Skin Temp	0.119	−0.413

**Table 10:** VIP Scores of Operational Variables Associated with Residual MFI Fluctuations.

Rank	Variable	VIP Score
1	Plate Fouling Factor	2.17
2	Production Rate	1.33
3	H_2_/C_3_ Ratio	1.17
4	Catalyst Productivity	0.96
5	Temp_for_Control	0.94
6	SCA/Ti Ratio	0.89
7	Distributor Plate DP	0.84
8	Cooler Fouling Factor	0.79
9	TEAL/Ti Ratio	0.71
10	TEAL/SCA Ratio	0.67
11	Reactor Pressure	0.54
12	Cycle Gas Density	0.48

**Table 11:** Pearson Correlation Coefficients between Operational Variables and MFI.

Variable	r
Temp_for_Control	0.166
Plate Fouling Factor	−0.162
SCA/Ti Ratio	0.141
Cooler Fouling Factor	−0.140
TEAL/Ti Ratio	0.115
Catalyst Productivity	−0.113
TEAL/SCA Ratio	−0.112

**Table 12:** Comparison of Key Findings with Literature and Industrial Practice.

Factor	Findings in This Work	Literature/Industrial Evidence	Agreement
H_2_/C_3_ Ratio	VIP = 1.17; positive contribution to PC2	Hydrogen acts as the primary chain-transfer agent controlling molecular weight and MFI in polypropylene polymerization.	Yes
SCA/Ti Ratio	Strong negative loading in PC2 (−0.419)	External donors strongly influence catalyst stereospecificity, hydrogen response, and molecular-weight development.	Yes
TEAL/SCA Ratio	Strong positive loading in PC2 (+0.434)	Donor/co-catalyst balance controls active-site distribution and polymer microstructure.	Yes
Catalyst Productivity	VIP = 0.96; loading in PC2 = 0.379	Catalyst activity is directly related to polymer molecular architecture and process performance.	Yes
Plate Fouling Factor	Highest VIP score (2.17)	Fouling and sheet formation affect heat transfer, gas circulation, and reactor stability.	Yes
Production Rate	VIP = 1.33	Changes in throughput affect the residence time distribution and thermal balance.	Yes
Temperature Control	Highest Pearson correlation (r = 0.166)	Reactor temperature affects propagation and chain-transfer kinetics.	Yes

## Data Availability

The original contributions presented in this study are included in the article/Supplementary Material. Further inquiries can be directed to the corresponding author.

## Supplementary Materials

The following supporting information can be downloaded at https://www.mdpi.com/article/10.3390/polym18131670/s1.
