# Supplementary material for: Operational Domains Governing Melt Flow Index Variability in Industrial Polypropylene Production
Source: Polymers (Basel). 2026 Jul 6;18(13):1670. doi: 10.3390/polym18131670 (PMC13364017; doi:10.3390/polym18131670)
Supplement: Supplementary file 1 [file polymers-18-01670-s001.zip › polymers-4407937-supplementary.pdf]

Supplementary Materials

This section corresponds to the supplementary material mentioned in the article document.

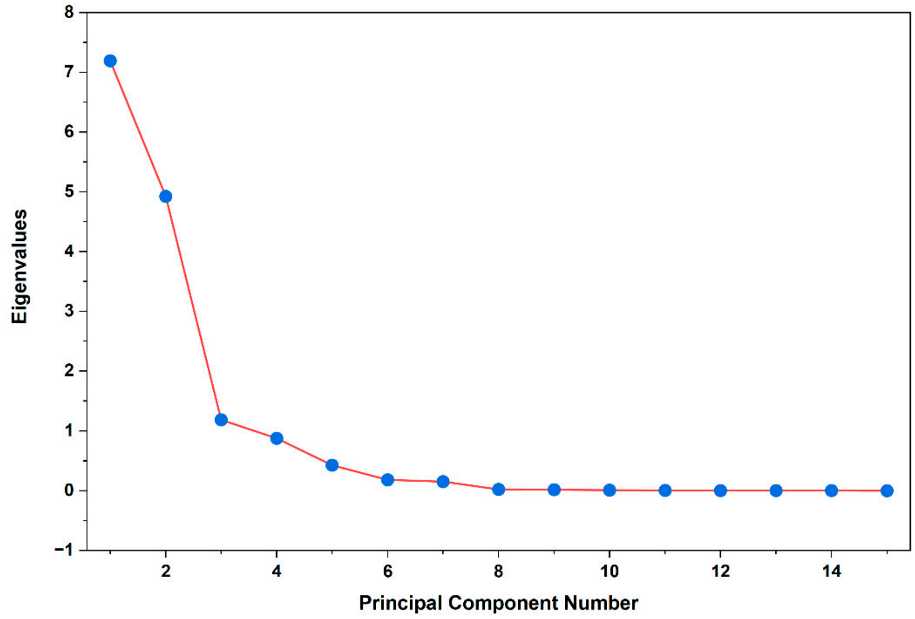

Figure S1. Scree plot of principal components.

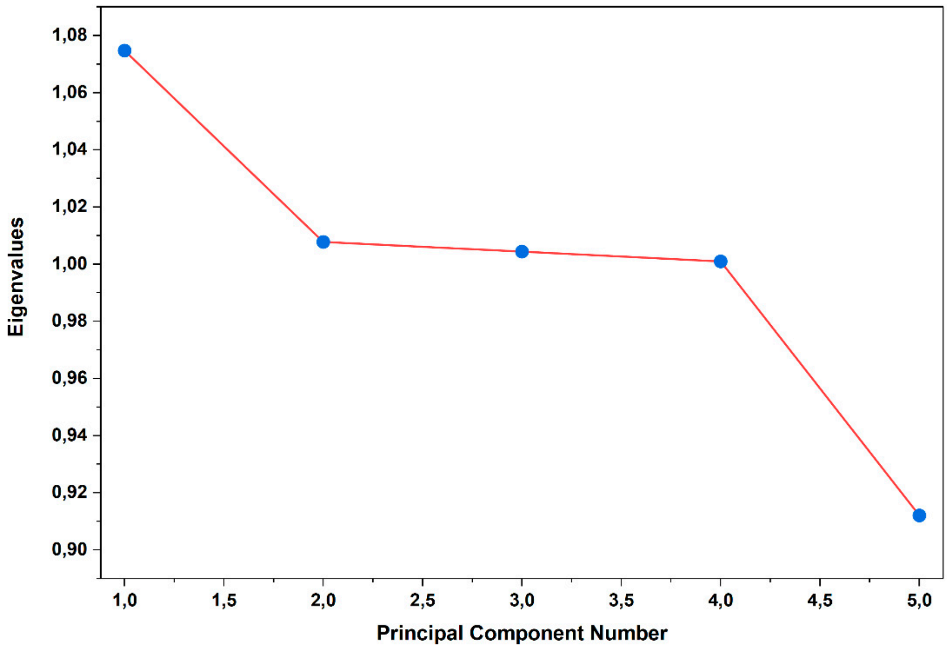

Figure S2. Cumulative explained variance.

Table S1. Principal Component Analysis (PCA) Validation Statistics.

Principal Component	Explained Variance (%)	Cumulative Variance (%)
PC1	51.35	51.35
PC2	35.14	86.49
PC3	6.85	93.34
PC4	3.55	96.89
PC5	1.36	98.25
PC6	1.30	99.55
PC7	0.16	99.71
PC8	0.15	99.86

Table S2. Complete Variable Importance in Projection (VIP) Ranking for Melt Flow Index Variability.

Rank	Variable	VIP Score
1	Plate Fouling Factor	2.167
2	Production Rate	1.327
3	H_2/C_3 Ratio	1.170
4	Catalyst Productivity	0.960
5	Temp_for_Control	0.940
6	SCA/Ti Ratio	0.890
7	Distributor Plate DP	0.840
8	Cooler Fouling Factor	0.790
9	TEAL/Ti Ratio	0.710
10	TEAL/SCA Ratio	0.670
11	Reactor Pressure	0.540
12	Cycle Gas Density	0.480
